# Overweight and Hypertension in Relation to Chronic Musculoskeletal Pain Among Community-Dwelling Adults: The Circulatory Risk in Communities Study (CIRCS)

**DOI:** 10.2188/jea.JE20200135

**Published:** 2021-11-05

**Authors:** Hironobu Kakihana, Hiroshige Jinnouchi, Akihiko Kitamura, Ko Matsudaira, Masahiko Kiyama, Mina Hayama-Terada, Isao Muraki, Yasuhiko Kubota, Kazumasa Yamagishi, Takeo Okada, Hironori Imano, Hiroyasu Iso

**Affiliations:** 1 Public Health, Department of Social Medicine, Osaka University Graduate School of Medicine, Osaka, Japan; 2 Department of Hygiene and Public Health, Nippon Medical School, Tokyo, Japan; 3 Osaka Center for Cancer and Cardiovascular Disease Prevention, Osaka, Japan; 4 Research Team for Social Participation and Community Health, Tokyo Metropolitan Institute of Gerontology, Tokyo, Japan; 5 Department of Medical Research and Management for Musculoskeletal Pain, 22nd Century Medical & Research Center, Faculty of Medicine, the University of Tokyo, Tokyo, Japan; 6 Yao City Public Health Center, Osaka, Japan; 7 Department of Public Health Medicine, Faculty of Medicine, and Health Services Research and Development Center, University of Tsukuba, Ibaraki, Japan

**Keywords:** back pain, knee pain, overweight, hypertension, cross-sectional study

## Abstract

**Background:**

The association between overweight and chronic musculoskeletal pain may vary by anatomical site and be modified by hypertension status. This study examined the associations between overweight and low back and knee pains and their effect modification by hypertension status.

**Methods:**

We conducted a community-based cross-sectional study involving 2,845 adults (1,080 men and 1,765 women) aged 40–89 years. Chronic knee pain (CKP) and low back pain (CLBP) lasting more than 3 months were categorized into more or less severe pain. Odds ratios (ORs) and 95% confidence intervals (CIs) of the association between overweight and more or less severe CKP and CLBP were determined using logistic regression and stratified by hypertension status. Adjustment variables were age, sex, area, hypertension, smoking and drinking status, inactivity, job category, mental stress, depression, and overall CKP or CLBP.

**Results:**

Overall, 288 (10.1%) and 631 (22.2%) adults had more and less severe CKP, respectively, and 284 (10.0%) and 830 (29.2%) had more and less severe CLBP, respectively. Overweight was associated with overall CKP and more or less severe CKP, regardless of hypertension status. Overweight was not associated with overall CLBP; its association was more pronounced for more severe CLBP. The association between overweight and more severe CLBP was evident among non-hypertensives (multivariable OR 1.72; 95% CI, 1.09–2.71); however, that between overweight and less severe CLBP was not evident (multivariable OR 1.07; 95% CI, 0.73–1.56).

**Conclusions:**

As hypertension may attenuate the association between overweight and CLBP, we should consider hypertension status for proper management of CLBP among overweight individuals.

## INTRODUCTION

Chronic musculoskeletal pain, represented by chronic knee pain (CKP) and chronic low back pain (CLBP), likely reduces social participation and activities of daily living.^1^^,^^2^ Overweight, defined as body mass index (BMI) of ≥25 kg/m^2^, has been considered to be associated with CKP and CLBP.^3^^–^^7^ A meta-analysis reported a positive association between overweight and CLBP; pooled odds ratio (OR) was 1.33 (95% confidence interval [CI], 1.14–1.56),^8^ but did not examine the severity of CLBP. In addition, previous studies investigated the association between overweight and knee or low back pain separately, without taking into consideration the coexistence of knee and low back pain, although they often coincide.^9^^,^^10^

A previous cross-sectional study of 17,128 men and women aged over 20 years reported that CLBP was less frequent among individuals with hypertension (defined as systolic blood pressure of ≥140 mm Hg and/or diastolic blood pressure of ≥90 mm Hg and/or antihypertensive medication use) compared to those without hypertension.^11^ In that study, systolic and diastolic blood pressures were inversely associated with CLBP, but the inverse associations of long-term hypertension and hypertension under medication use with CLBP were not observed.^11^ Another cross-sectional study of 46,901 men and women aged 30 and older reported that higher systolic and diastolic blood pressures were inversely associated with the prevalence of chronic musculoskeletal pain and that increased blood pressure levels during the past 11 years were also inversely associated with the prevalence of pain.^12^ These results support a hypertension-induced analgesia theory.^13^ Although hypertension is likely to be prevalent in overweight individuals, the effect of hypertension on the association between overweight and musculoskeletal pain is unknown. For proper management of musculoskeletal pain, it is necessary to evaluate the association between overweight and musculoskeletal pain by the presence or absence of hypertension.

The aim of this study was to examine the association between overweight and chronic musculoskeletal pain considering pain severity, coexistence of knee and low back pain, and an effect modification by hypertension status among community-dwelling adults. We hypothesized that overweight would be associated with CKP and CLBP, and that the associations of overweight with CKP and CLBP would be more pronounced in non-hypertensive individuals than in hypertensive individuals.

## METHODS

### Study population

The study population included residents from Ikawa (a rural community in the Akita Prefecture of northeastern Japan) and Minami-Takayasu (a suburban of the Osaka Prefecture in mid-western Japan) communities who were enrolled in the Circulatory Risk in Communities Study (CIRCS).^14^^,^^15^ This study was conducted in 2016 and 2,970 residents aged 40–89 years participated in it. We excluded participants who did not fully complete the questionnaire (*n* = 123), as well as those lacking data on blood pressure (*n* = 1) and BMI (*n* = 1). Finally, data from 2,845 participants (1,080 men and 1,765 women) were analyzed. This study was approved by the ethics committees of the Osaka Center for Cancer and Cardiovascular Disease Prevention (27-ethics-2) and Osaka University (14285).

### Outcome variables

CKP and CLBP were assessed using a self-administered questionnaire with an illustration showing the area of pain according to the recommendations of Dionne et al^16^ and the International Association for the Study of Pain.^17^ If a participant had experienced pain in the past 4 weeks and the pain had continued for 3 or more months, we defined that pain as CKP or CLBP.^16^^,^^17^ When the participants had experienced chronic pain, the severity of pain was classified according to the following questions: “Is this pain enough to limit your daily activities such as walking, stair climbing, sitting, lying and carrying luggage?”. If the answer was “yes”, we regarded the pain as more severe, and as less severe if the answer was “no”. In our subsamples of 44 individuals with CKP and 52 individuals with CLBP, we assessed the severity of pain using the standardized tools: the Knee Injury and Osteoarthritis Outcome Score subscales for CKP (KOOS_4_: covering pain, symptoms, activities of daily living, and quality of life),^18^^–^^20^ and Roland-Morris Disability Questionnaire for CLBP (RDQ).^21^^,^^22^ Participants who reported more severe pain had the worse (higher) scores of KOOS_4_ and RDQ than those with less severe pain; mean score for KOOS_4_ was 63.7 (standard deviation [SD], 12.4) versus 75.4 (SD, 12.4) (*P* = 0.004), and that for RDQ was 6.9 (SD, 4.0) versus 3.5 (SD, 3.5) (*P* = 0.003).

### Explanatory variables

Height was measured wearing socks. Weight was measured in light clothing, and the value by subtracting 1 kg as the clothing weight was recorded. We calculated BMI as body weight in kilograms (kg) divided by height in meters (m) squared. Overweight was defined as BMI ≥25 kg/m^2^ based on the criteria of the Japan Society of Obesity.^23^ Blood pressure was measured by trained physicians using the mercury sphygmomanometers on right upper arm after the participants rested for 5 minutes. We defined hypertension as systolic blood pressure (SBP) ≥140 mm Hg and/or diastolic blood pressure (DBP) ≥90 mm Hg, and/or antihypertensive medication use. Trained interviewers collected information on smoking status, drinking habit, physical activity, mental stress, current job, and depressive state. Participants who smoked ≥1 cigarette per day were classified as current smokers, and those who had quit smoking as past smokers, while those who reported drinking one or more times per week were considered as current drinkers and those who had quit drinking as ex-drinkers. We regarded those who answered “no” to all of the following three questions as inactivity: 1) “Have you exercised to sweat for 30 minutes or longer at least twice a week for more than a year?”, 2) “Compared with individuals of the similar age and same sex, do you walk faster?”, and 3) “Do you walk or do similar physical activities for an hour or more per day?” Moreover, based on the following question: “Do you feel stressed about work or everyday life?”, we classified the mental stress state into three categories “not at all”, “a little”, and “high or extremely high”. Additionally, when the answer to both the following two questions were “yes”, they were regarded to be in a state of depression: 1) “Have you had little or no interest or enjoyment in anything you do for the past month?” and 2) “Have you been feeling depressed and hopeless for the past month?”. The current job was regrouped from the Japan Standard Occupational Classification (Rev. December 5, 2009) into seven categories (manager, administrator, or professional worker, office worker, retail or service industry worker, agriculture or fisheries worker, machine manufacturer or operator, manual worker, and unemployed) as done in a previous study.^11^

### Statistical analysis

Differences in the characteristics of participants according to overweight status were examined using chi-square test. To examine the association between blood pressure categories and CKP and CLBP, we categorized into four levels of blood pressures (non-hypertensives: SBP <140 mm Hg and DBP <90 mm Hg and no antihypertensive medication use; controlled hypertensives: SBP <140 mm Hg and DBP <90 mm Hg and antihypertensive medication use; moderate hypertensives: 140≤ SBP <160 mm Hg and/or 90≤ DBP <100 mm Hg and/or antihypertensive medication use; severe hypertensives: SBP ≥160 mm Hg and/or DBP ≥100 mm Hg and/or antihypertensive medication use). We calculated the ORs and 95% CIs for overall CKP or CLBP associated with hypertension and overweight via logistic regression analysis. Moreover, we calculated the OR and 95% CIs for more or less severe CKP or CLBP using multinomial logistic regression analysis to consider the severity of pain. The possible confounding variables for multivariable adjustment included age, sex, hypertension, smoking status (current, past, and never), drinking status (current, past, and never), inactivity (yes or no), mental stress (not at all, a little, and high or extremely high), depression (yes or no), area (rural or urban), and current job (seven categories mentioned above). We also adjusted for overall CKP or CLBP mutually because CKP and CLBP often coexist.^9^^,^^10^ We performed a stratified analysis by presence or absence of hypertension to examine whether the associations between overweight and CKP or CLBP were modified by hypertension status. The test for effect modification by hypertension status was conducted with an interaction term generated by multiplying overweight by hypertension status.

Statistical analyses were performed with the SAS9.4 (SAS Institute Inc., Cary, NC, USA). Two-tailed *P* values of <0.05 were considered as statistically significant.

## RESULTS

The characteristics of the participants according to their overweight status are summarized in Table 1. Among 2,845 participants (1,080 men and 1,765 women), 631 had less severe CKP (22.2%), 288 had more severe CKP (10.1%), 830 had less severe CLBP (29.2%), and 284 had more severe CLBP (10.0%).

**Table 1.** Characteristics of 2,845 study participants according to the presence or absence of overweight

	Total	Overweight^a^		*P*-value

		No	Yes	
Number of participants	2,845	2,038	807	
Age, years, %				
40–49	12.3	12.7	11.3	0.37
50–59	13.3	13.9	11.8	
60–69	34.5	33.8	36.3	
70–79	31.5	31.4	31.8	
80–89	8.4	8.2	8.8	
Women, %	62.0	65.5	53.3	<0.001
Hypertension^b^, %	52.3	44.7	71.3	<0.001
Smoking status, %				
Never	59.8	61.6	55.1	0.004
Past	27.3	25.8	31.4	
Current	12.9	12.6	13.5	
Drinking status, %				
Never	48.7	49.5	46.6	0.35
Past	10.3	10.3	10.4	
Current	41.0	40.2	43.0	
Inactivity, %	28.0	24.8	36.2	<0.001
Job, %				
Manager, administrator, or professional worker	5.6	5.6	5.6	0.001
Office worker	5.4	5.7	4.8	
Retail or service industry worker	11.9	12.6	10.2	
Agriculture or fisheries worker	9.9	8.6	13.1	
Machine manufacturer or operator	2.8	2.5	3.6	
Manual worker	10.2	9.8	11.4	
Unemployed	54.1	55.2	51.3	
Mental stress, %				
Not at all	28.0	26.5	31.8	0.01
A little	55.6	57.3	51.4	
High or extremely high	16.3	16.1	16.7	
Depression, %	6.4	6.7	5.7	0.61
Chronic knee pain (CKP), %				
No	67.7	70.4	60.8	<0.001
Less severe^c^	22.2	21.4	24.2	
More severe^d^	10.1	8.2	15.0	
Chronic low back pain (CLBP), %				
No	60.8	62.0	57.9	0.001
Less severe^c^	29.2	29.0	29.6	
More severe^d^	10.0	9.0	12.5	

^a^Body mass index ≥25.0 kg/m^2^.

^b^Systolic blood pressure ≥140 mm Hg and/or diastolic blood pressure ≥90 mm Hg and/or antihypertensive medication use.

^c^Pain with no interference to daily activities, such as walking difficulty.

^d^Pain that interferes with the daily activities.

Table 2 shows the ORs of overall chronic knee and low back pains according to hypertensive status. There were inverse associations between hypertensive status and CLBP but not CKP. The multivariable OR of CLBP for severe hypertensives versus non-hypertensives was 0.70 (95% CI, 0.49–0.99; *P* for trend = 0.09). There was no effect-modifications by age group (40–59 years and 60–89 years) for the association between hypertension and CLBP or CKP (*P* for interaction 0.76 for CKP, 0.95 for CLBP, not shown in Table).

**Table 2.** Odds ratios (95% confidence intervals) for overall chronic knee and low back pain according to hypertensive status

	Blood pressure category					*P*-value for trend^d^

	Non-hypertensives^a^	Hypertensives	Hypertensives			

			SBP <140 mm Hg and DBP <90 mm Hg and antihypertensive medication use	140≤ SBP <160 mm Hg and/or 90≤ DBP <100 mm Hg and/or antihypertensive medication use	SBP ≥160 mm Hg and/or DBP ≥100 mm Hg and/or antihypertensive medication use	
Number at risk	1,358	1,487	658	646	183	
Overall CKP						
Number of case	400	519	254	216	49	
Age-, sex- and area-adjusted OR	1.00	1.18 (1.00–1.40)	1.32 (1.07–1.63)^†^	1.14 (0.93–1.41)	0.91 (0.63–1.29)	0.57
Multivariable OR^b^	1.00	1.05 (0.88–1.26)	1.15 (0.93–1.43)	1.04 (0.84–1.29)	0.80 (0.55–1.14)	0.62
Multivariable OR^c^	1.00	1.08 (0.89–1.29)	1.14 (0.91–1.43)	1.07 (0.85–1.33)	0.89 (0.60–1.30)	0.99
Overall CLBP						
Number of case	523	591	280	252	59	
Age-, sex- and area-adjusted OR	1.00	1.01 (0.86–1.19)	1.14 (0.93–1.39)	0.99 (0.81–1.21)	0.73 (0.52–1.02)	0.25
Multivariable OR^b^	1.00	0.94 (0.80–1.12)	1.05 (0.85–1.29)	0.94 (0.77–1.15)	0.68 (0.48–0.95)^*^	0.08
Multivariable OR^c^	1.00	0.93 (0.78–1.10)	1.01 (0.81–1.25)	0.93 (0.75–1.15)	0.70 (0.49–0.99)^*^	0.09

CKP, chronic knee pain; CLBP, chronic low back pain; DBP, diastolic blood pressure; OR, odds ratio; SBP, systolic blood pressure.

^*^P < 0.05, ^†^*P* < 0.01.

^a^SBP <140 mm Hg and DBP <90 mm Hg and no antihypertensive medication use.

^b^Adjusted for age, sex, area, overweight, inactivity, smoking status, drinking status, mental stress, depressive status, and job.

^c^Adjusted further for overall CLBP or CKP.

^d^Odds ratios per 1 increase blood pressure category for overall CKP or CLBP.

Table 3 shows the ORs of the associations between overweight and overall CKP and CLBP in the total population and in the population stratified by hypertension status. Overweight was significantly associated with overall CKP even after adjusting for potential confounders (OR 1.61; 95% CI, 1.34–1.94, *P* < 0.001) and after further adjustment for overall CLBP (OR 1.59; 95% CI, 1.31–1.92, *P* < 0.001). The association between overweight and overall CKP did not vary by hypertension status. In contrast, overweight tended to be associated with overall CLBP, but the association was weakened after adjusting for overall CKP (before adjustment: multivariable adjusted OR 1.18; 95% CI, 0.99–1.41, *P* = 0.07; after adjustment for overall CKP: OR 1.04; 95% CI, 0.86–1.25, *P* = 0.72). These ORs were slightly greater in non-hypertensive individuals than in hypertensive individuals, but no significant interaction effect of overweight and hypertension was found in both groups for overall CKP and CLBP.

**Table 3.** Odds ratios of overall chronic knee pain and chronic low back pain associated with overweight compared to non-overweight in total participants and stratified by hypertensive status

	Total		Non-hypertensives		Hypertensives^a^		*P*-value for interaction^e^

	Non-overweight	Overweight^b^	Non-overweight	Overweight^b^	Non-overweight	Overweight^b^	
Number at risk	2,038	807	1,126	232	912	575	
Overall CKP							
Number of case	603	316	313	87	290	229	
Age-, sex-, and area-adjusted OR	1.00	1.64 (1.38–1.96)^‡^	1.00	1.70 (1.25–2.30)^‡^	1.00	1.57 (1.25–1.96)^‡^	0.75
Multivariable OR^c^	1.00	1.61 (1.34–1.94)^‡^	1.00	1.73 (1.26–2.36)^‡^	1.00	1.57 (1.25–1.97)^‡^	0.82
Multivariable OR^d^	1.00	1.59 (1.31–1.92)^‡^	1.00	1.65 (1.19–2.28)^†^	1.00	1.55 (1.22–1.98)^‡^	0.97
Overall CLBP							
Number of case	774	340	421	102	353	238	
Age-, sex-, and area-adjusted OR	1.00	1.19 (1.00–1.40)^*^	1.00	1.31 (0.98–1.75)	1.00	1.14 (0.92–1.42)	0.44
Multivariable OR^c^	1.00	1.18 (0.99–1.41)	1.00	1.35 (1.00–1.81)^*^	1.00	1.15 (0.92–1.43)	0.45
Multivariable OR^d^	1.00	1.04 (0.86–1.25)	1.00	1.20 (0.88–1.63)	1.00	1.00 (0.79–1.27)	0.45

CKP, chronic knee pain; CLBP, chronic low back pain; OR, odds ratio.

^*^P < 0.05, ^†^*P* < 0.01, ^‡^*P* < 0.001.

^a^Systolic blood pressure ≥140 mm Hg and/or diastolic blood pressure ≥90 mm Hg and/or antihypertensive medication use.

^b^Body mass index ≥25.0 kg/m^2^.

^c^Adjusted for age, sex, area, hypertension, inactivity, smoking status, drinking status, mental stress, depressive status, and job.

^d^Adjusted further for overall CLBP or CKP.

^e^Interaction between overweight and hypertension status.

Table 4 shows the ORs of the associations between overweight and more or less severe CKP and CLBP in the total population and in the population stratified by hypertension status. We observed a stronger association between overweight and more severe CKP than less severe CKP (the multivariable OR of more severe CKP was 2.24; 95% CI, 1.69–2.97, *P* < 0.001; after further adjustment for overall CLBP: OR 2.19; 95% CI, 1.64–2.92, *P* < 0.001; the multivariable OR of less severe CKP was 1.39; 95% CI, 1.13–1.71, *P* = 0.002; after further adjustment for overall CLBP: OR 1.37; 95% CI, 1.10–1.70, *P* = 0.004). These associations did not vary by hypertension status. However, a significant association was found only between overweight and more severe CLBP, but not after adjusting for overall CKP. Moreover, the association was more pronounced in non-hypertensive than in hypertensive individuals (the multivariable OR after adjustment for confounding variables was 1.96; 95% CI, 1.26–3.05, *P* = 0.003; after further adjustment for overall CKP: OR 1.72; 95% CI, 1.09–2.71, *P* = 0.02 among non-hypertensives; and OR 1.23; 95% CI, 0.85–1.78, *P* = 0.28 and OR 1.07; 95% CI, 0.73–1.56, *P* = 0.74, respectively, among hypertensives). The interaction for the association between overweight and more severe CLBP by hypertensive status was statistically significant (*P* for interaction = 0.046).

**Table 4.** Odds ratios of more/less severe chronic knee pain and chronic low back pain associated with overweight compared to non-overweight in total participants and stratified by hypertensive status

	Total		Non-hypertensives		Hypertensives^a^		*P*-value for interaction^e^

	Non-overweight	Overweight^b^	Non-overweight	Overweight^b^	Non-overweight	Overweight^b^	
Number at risk	2,038	807	1,126	232	912	575	
Less severe CKP							
Number of case	436	195	237	56	199	139	
Age-, sex-, and area-adjusted OR	1.00	1.39 (1.13–1.70)^†^	1.00	1.42 (1.00–2.01)^*^	1.00	1.35 (1.04–1.74)^*^	0.90
Multivariable OR^c^	1.00	1.39 (1.13–1.71)^†^	1.00	1.45 (1.02–2.07)^*^	1.00	1.36 (1.05–1.78)^*^	0.92
Multivariable OR^d^	1.00	1.37 (1.10–1.70)^†^	1.00	1.38 (0.96–2.00)	1.00	1.36 (1.03–1.79)^*^	0.93
More severe CKP							
Number of case	167	121	76	31	91	90	
Age-, sex-, and area-adjusted OR	1.00	2.34 (1.79–3.06)^‡^	1.00	2.65 (1.65–4.27)^‡^	1.00	2.08 (1.49–2.89)^‡^	0.48
Multivariable OR^c^	1.00	2.24 (1.69–2.97)^‡^	1.00	2.72 (1.67–4.43)^‡^	1.00	2.05 (1.45–2.89)^‡^	0.52
Multivariable OR^d^	1.00	2.19 (1.64–2.92)^‡^	1.00	2.61 (1.59–4.28)^‡^	1.00	2.02 (1.42–2.88)^‡^	0.61
Less severe CLBP							
Number of case	591	239	329	64	262	175	
Age-, sex-, and area-adjusted OR	1.00	1.09 (0.91–1.32)	1.00	1.08 (0.77–1.50)	1.00	1.11 (0.88–1.41)	0.75
Multivariable OR^c^	1.00	1.10 (0.91–1.34)	1.00	1.15 (0.82–1.61)	1.00	1.12 (0.87–1.43)	0.84
Multivariable OR^d^	1.00	0.97 (0.79–1.19)	1.00	1.03 (0.72–1.45)	1.00	0.98 (0.75–1.26)	0.88
More severe CLBP							
Number of case	183	101	92	38	91	63	
Age-, sex-, and area-adjusted OR	1.00	1.49 (1.14–1.95)^†^	1.00	2.08 (1.35–3.19)^†^	1.00	1.23 (0.86–1.76)	0.02
Multivariable OR^c^	1.00	1.43 (1.08–1.90)^*^	1.00	1.96 (1.26–3.05)^†^	1.00	1.23 (0.85–1.78)	0.04
Multivariable OR^d^	1.00	1.25 (0.93–1.67)	1.00	1.72 (1.09–2.71)^*^	1.00	1.07 (0.73–1.56)	0.046

CKP, chronic knee pain; CLBP, chronic low back pain; OR, odds ratio.

^*^P < 0.05, ^†^*P* < 0.01, ^‡^*P* < 0.001.

^a^Systolic blood pressure ≥140 mm Hg and/or diastolic blood pressure ≥90 mm Hg and/or antihypertensive medication use.

^b^Body mass index ≥25.0 kg/m^2^.

^c^Adjusted for age, sex, area, hypertension, inactivity, smoking status, drinking status, mental stress, depressive status, and job.

^d^Adjusted further for overall CLBP or CKP.

^e^Interaction between overweight and hypertension status.

## DISCUSSION

In this community-based cross-sectional study, overweight showed a significant positive association with overall CKP even after adjustment for overall CLBP, although the association was stronger for more severe CKP than for less severe CKP. These significant associations remained unchanged regardless of hypertension status. Moreover, a weak and non-significant association was observed between overweight and overall CLBP after adjustment for overall CKP, although the association was more pronounced for more severe CLBP. The association between overweight and more severe CLBP was more evident in non-hypertensive individuals than in hypertensive individuals.

Previous studies examined the association between overweight and knee pain^3^^,^^6^^,^^7^ and found that the strength of association varied according to the degree of overweight and the severity of knee pain.^6^ A cross-sectional study of 576 American men and women aged ≥40 years showed a positive association between overweight and knee pain; the multivariable OR of knee pain was 1.6 (95% CI, 1.0–2.5) for BMI 25.0–29.9 kg/m^2^ after adjusting for age, sex, and osteoarthritis grade using the criteria of Kellgeren & Lawrence.^7^ Another cross-sectional study^6^ involving 4,515 British men and women aged ≥16 years showed a positive association between BMI and knee pain in dose-response manner (the multivariable ORs were 1.51; 95% CI, 1.23–1.86 for BMI 25–29.9 kg/m^2^ and 2.06; 95% CI, 1.56–2.73 for BMI ≥30 kg/m^2^ with reference to BMI 20.0–24.9 kg/m^2^) after adjusting for age, sex, socioeconomic status, and other site pain. In addition, a stronger association was observed in more severe knee pain; the multivariable ORs were 1.98 (95% CI, 1.44–2.73) for BMI 25–29.9 kg/m^2^ and 3.25 (95% CI, 2.21–4.77) for BMI ≥30 kg/m^2^. Our results are consistent with the findings from the American and British cross-sectional studies.

Previous studies also showed a positive association between overweight and low back pain^4^^,^^5^ and the association became stronger as BMI increased.^4^^,^^5^ However, past studies did not investigate the association between overweight and pain severity. The Nord-Trøndelag Health Study involving 63,968 Norwegian men and women aged ≥20 years showed a positive association between higher BMI and CLBP^4^; the multivariable ORs of CLBP in reference to BMI 20–24.9 kg/m^2^ were 1.06 (95% CI, 0.99–1.25) for BMI 25–29.9 kg/m^2^, 1.13 (95% CI, 1.02–1.25) for BMI 30–34.9 kg/m^2^, and 1.30 (95% CI, 1.07–1.58) for BMI ≥35 kg/m^2^ in men; and in women, respective ORs were 1.22 (95% CI, 1.14–1.30), 1.45 (95% CI, 1.33–1.59), and 1.70 (95% CI, 1.52–1.90) for each BMI category, after adjusting for age, education, smoking, leisure time physical activity, work status, and physical activity at work. As 86% of our overweight participants had BMI 25–29.9 kg/m^2^, our multivariable OR of CLBP for overweight of 1.18 (95% CI, 0.99–1.41) is similar to estimates reported in previous studies for the same BMI range.^4^^,^^5^ However, taking into account the coexistence of CKP, which was not considered in previous studies, the association between overweight and overall CLBP disappeared. This study is the first to show that hypertension modifies the association between overweight and more severe CLBP.

In our study, overweight was more strongly associated with CKP than CLBP, which was consistent with the findings from previous studies in Western countries.^4^^–^^7^ A previous study reported an association between overweight and osteoarthritis (including asymptomatic). A cross-sectional study of 1,118 Korean men and women aged ≥65 years showed that overweight was more strongly associated with knee osteoarthritis than with lumbar osteoarthritis; the multivariable ORs of knee osteoarthritis and lumbar osteoarthritis for BMI ≥25.0 kg/m^2^ after adjustment for age and sex were 3.4 (95% CI, 2.4–5.0) and 1.5 (95% CI, 1.1–2.2), respectively.^24^

Mechanical stress exerted through the body weight has been suggested as a major cause of musculoskeletal pain,^4^^,^^5^ and body weight generally generates greater load on the knee joint than on the lumbar spine.^25^^,^^26^ In case of walking, the estimated load on the knee joint is 2.5 times the body weight,^25^ while that on the lumber spine is around one’s body weight.^26^ Our results, as well as the findings from previous studies^25^^,^^26^ that overweight showed a stronger association with knee pain than with low back pain can be explained by the load mechanisms.

Considering the results of our analysis, hypertension-induced analgesia is another possible mechanism involved in our results. Hypertension has been suggested to inhibit spinal pain transmission via baroreflex system,^13^ although some of previous studies with small sample sizes (range, *n* = 56 to *n* = 95) and no adjustment for important confounding factors, such as age, suggested that elevated blood pressure is associated with increased pain sensitivity among individuals with chronic pain.^27^^,^^28^ Our result that overweight had a non-significant association with more severe CLBP among hypertensive individuals was consistent with the hypertension-induced analgesia theory. A cross-sectional study suggested that long-term hypertension, which was generally observed in older adults, may reduce the effect of hypertension-induced analgesia for CLBP.^11^ In our study, however, we did not find the age interaction in the association between hypertension and CLBP. Since the present study suggests that hypertension attenuate the association between overweight and CLBP, monitoring CLBP among hypertensive persons may be necessary. Lack or weakened pain, which is a warning function against noxious stimuli, may cause prolongation and/or advancement of CLBP.

The strength of the present study includes its population-based design to systematically evaluate the associations between overweight and more or less severe CLBP and CKP. Our study is the first to show that hypertension status modifies the association between overweight and CLBP. However, our study has some limitations. First, as this was a cross-sectional study, we were unable to establish any causal relationships. Second, like the previous studies, we did not consider the length of time that participants had been overweight. It may be necessary to conduct a study considering the cumulative period of overweight in the future.

In conclusion, overweight was associated with overall CKP and more or less severe CKP regardless of hypertension status. The association between overweight and more severe CLBP was more pronounced in non-hypertensive individuals than in hypertensive individuals, even after considering the coexistence of overall CKP. As hypertension may attenuate the association between overweight and CLBP, we need to pay attention to hypertension status to ensure optimal treatment and management of CLBP among overweight individuals.
